# DNA Methylation of *FKBP5* as Predictor of Overall Survival in Malignant Pleural Mesothelioma

**DOI:** 10.3390/cancers12113470

**Published:** 2020-11-21

**Authors:** Giovanni Cugliari, Chiara Catalano, Simonetta Guarrera, Alessandra Allione, Elisabetta Casalone, Alessia Russo, Federica Grosso, Daniela Ferrante, Clara Viberti, Anna Aspesi, Marika Sculco, Chiara Pirazzini, Roberta Libener, Dario Mirabelli, Corrado Magnani, Irma Dianzani, Giuseppe Matullo

**Affiliations:** 1 Department of Medical Sciences, University of Turin, 10126 Turin, Italy; chiara.catalano@unito.it (C.C.); alessandra.allione@unito.it (A.A.); elisabetta.casalone@unito.it (E.C.); alessia.russo@unito.it (A.R.); clara.viberti@unito.it (C.V.); 2 Italian Institute for Genomic Medicine, IIGM, 10060 Candiolo, Italy; simonetta.guarrera@iigm.it; 3 Candiolo Cancer Institute, FPO-IRCCS, 10060 Candiolo, Italy; 4 Division of Medical Oncology, SS. Antonio e Biagio General Hospital, 15121 Alessandria, Italy; federica.grosso@unipo.it; 5 Unit of Medical Statistics, Department of Translational Medicine, University of Piemonte Orientale, 28100 Novara, Italy; daniela.ferrante@med.uniupo.it (D.F.); corrado.magnani@med.uniupo.it (C.M.); 6 Cancer Epidemiology Unit, CPO-Piemonte, 28100 Novara, Italy; 7 Department of Health Sciences, University of Piemonte Orientale, 28100 Novara, Italy; anna.aspesi@med.uniupo.it (A.A.); marika.sculco@med.uniupo.it (M.S.); irma.dianzani@med.unipmn.it (I.D.); 8 Department of Experimental, Diagnostic and Specialty Medicine (DIMES), University of Bologna, 40126 Bologna, Italy; chiara.pirazzini5@unibo.it; 9 Pathology Unit, SS. Antonio e Biagio General Hospital, 15122 Alessandria, Italy; rlibener@ospedale.al.it; 10 Cancer Epidemiology Unit, Department of Medical Sciences, University of Turin, 10126 Turin, Italy; dario.mirabelli@gmail.com; 11 Interdepartmental Center for Studies on Asbestos and Other Toxic Particulates “G. Scansetti”, University of Turin, 10126 Turin, Italy; 12 Medical Genetics Unit, AOU Città della Salute e della Scienza, 10126 Turin, Italy

**Keywords:** malignant pleural mesothelioma, asbestos exposure, DNA methylation, lymphocyte-to-monocyte ratio, epigenome-wide analysis, survival analysis

## Abstract

**Simple Summary:**

Our study is the first one to investigate DNA methylation changes in white blood cells (WBCs) from easily accessible peripheral blood as malignant pleural mesothelioma (MPM) survival biomarker. The Cox proportional hazards regression model highlighted that the methylation status of the CpG dinucleotide cg03546163 is an independent marker of prognosis in MPM patients with a better performance than traditional inflammation-based scores such as lymphocyte-to-monocyte ratio (LMR). Biological validation and replication showed that epigenetic changes at the *FKBP5* gene were robustly associated with overall survival (OS) in MPM cases. The identification of simple and valuable prognostic markers for MPM will enable clinicians to select patients who are most likely to benefit from aggressive therapies and avoid subjecting non-responder patients to ineffective treatment.

**Abstract:**

Malignant pleural mesothelioma (MPM) is an aggressive tumor with median survival of 12 months and limited effective treatments. The scope of this study was to study the relationship between blood DNA methylation (DNAm) and overall survival (OS) aiming at a noninvasive prognostic test. We investigated a cohort of 159 incident asbestos exposed MPM cases enrolled in an Italian area with high incidence of mesothelioma. Considering 12 months as a cut-off for OS, epigenome-wide association study (EWAS) revealed statistically significant (*p* value = 7.7 × 10^−9^) OS-related differential methylation of a single-CpG (cg03546163), located in the 5′UTR region of the *FKBP5* gene. This is an independent marker of prognosis in MPM patients with a better performance than traditional inflammation-based scores such as lymphocyte-to-monocyte ratio (LMR). Cases with DNAm < 0.45 at the cg03546163 had significantly poor survival compared with those showing DNAm ≥ 0.45 (mean: 243 versus 534 days; *p* value< 0.001). Epigenetic changes at the *FKBP5* gene were robustly associated with OS in MPM cases. Our results showed that blood DNA methylation levels could be promising and dynamic prognostic biomarkers in MPM.

## 1. Introduction

Malignant pleural mesothelioma (MPM) is an aggressive tumor. The disease usually develops after a long latency (20–40 years) following asbestos exposure [1]. Although MPM is considered a rare malignancy (prevalence 1–9/100,000), about 40,000 deaths have been estimated to occur each year globally [2,3]. The World Health Organization estimates that 125 million people annually around the world are exposed to asbestos. The International Agency for Research on Cancer confirmed that all fibrous forms of asbestos are carcinogenic to humans, causing mainly mesothelioma, respiratory-tract tumors, mesothelioma, and cancer at other tissue sites [4].

The prognosis of MPM is poor with a median survival of about 12 months from the diagnosis [5].

Generally, the first-line treatment is a combination of a multitargeted anti folate (pemetrexed or raltitrexed) drug and a platinum compound (cisplatin or carboplatin) [6] Currently, only a single randomized trial demonstrated an increase in survival time when comparing cisplatin and pemetrexed versus cisplatin alone [7]; unfortunately, most patients became resistant to this treatment and relapsed rapidly. No oncogenic driver has been identified and molecular pathways leading to MPM have not yet been clearly determined. Other therapeutic strategies such as immunotherapy are promising but require further investigation and improvement [8].

Recent research on the pathogenesis of MPM indicated that (i) both genetic and epigenetic alterations contribute to asbestos-induced tumorigenesis [9,10], (ii) inflammation-based prognostic scores that include lymphocyte counts are associated with survival [11].

MPM has a low frequency of protein-altering mutations (~25 mutations per tumor), compared to many other tumors [12]. Moreover, germline mutations in different genes mainly involved in DNA damage repair confer moderate-to-high genetic risk of MPM development [13]. The BAP1-tumor predisposition syndrome is the most studied genetic condition associated with MPM development and is caused by mutations in the BRCA1-associated protein 1 (*BAP1*) gene [13].

In the last 10 years, epigenetic markers, such as DNA methylation (DNAm) and microRNAs (miRNAs), have gained popularity as possible early diagnostic and prognostic biomarkers in cancer research, including MPM. While genetic markers may differ from case to case in most cancer patients (i.e., each patient may carry a different mutation within the same gene), different subjects show variable levels of epigenetic biomarkers in specific target regions and different tissues depending on disease status [14].

DNA methylation is one of the epigenetic factors [15] that can be altered in cancer tissues. However, regarding mechanisms and clinical outcome of epigenetic derangements in MPM, less information is available [16,17] Although DNAm is stable, it can be modified throughout life by several factors such as ageing, lifestyle, environmental exposures, and diseases. It thus represents an adaptive phenomenon linking environmental factors and the development of pathologic phenotypes such as cancers. DNAm changes are considered to possibly play a role in MPM progression, and have therefore been suggested as a potential tool for prognosis [18].

The fact that epigenetic modifications, unlike genetic changes, are potentially reversible, may open new perspectives for patient clustering and novel therapeutic options. A reliable prognostic biomarker that offers high sensitivity and specificity would be a major advancement for MPM. Blood-based biomarkers that have been explored in MPM include megakaryocyte potentiating factor (an alternative cleavage product of the mesothelin precursor protein) [19], and Fibulin 3 which is also found in pleural fluid, and whose high levels appear to correlate with advanced disease [20].

Considering clinical end-point, low pleural fluid glucose and high C-reactive protein and pleural thickening represent the main prognosis factors [21]. Recent studies confirm that using also a combination of epigenetic alterations as biomarkers is more informative with respect to an only genetic approach on overall survival (OS) [17].

This study was undertaken with the goal of better characterizing the MPM OS evaluating the potential predictive value of peripheral blood DNAm profiles. The second goal was the comparison of the DNAm prognostic performance with the broadly used lymphocyte-to-monocyte ratio (LMR) method.

## 2. Results

### 2.1. Epigenome-Wide Association Study (EWAS)

**Figure 1:**
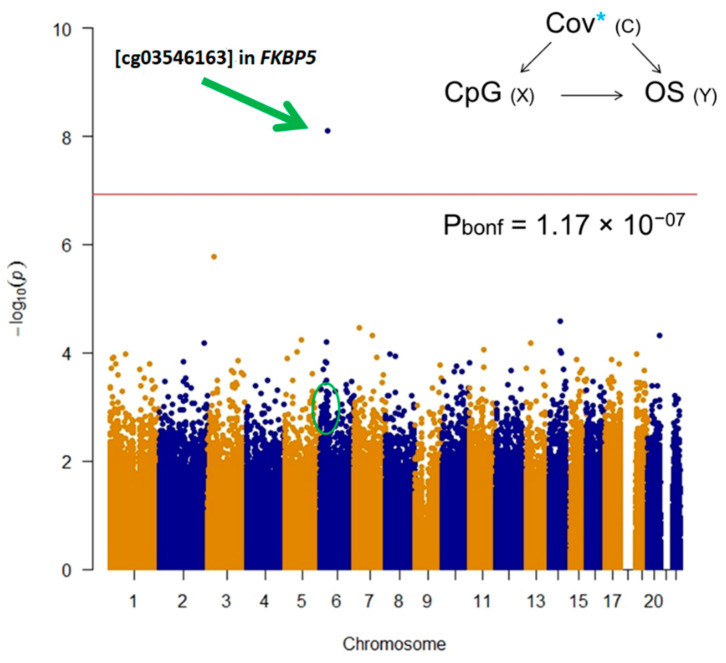
Manhattan plot for epigenome-wide association study (EWAS) test on 450k single CpGs. Overall survival was used as dependent variable considering 12 months as cut-off adjusting for age, gender, histological subtype, asbestos exposure, WBCs estimation, population stratification, and technical variability. Bonferroni post hoc line highlights statistically significant differences on OS at single CpG level.

EWAS revealed a statistically significant hypo-methylated single-CpG (cg03546163) in the *FKBP5* gene in the low survival group after Bonferroni post-hoc correction (Figure 1).

Bootstrap was computed to estimate the measures of accuracy, using random sampling methods.

The other five CpGs in the *FKBP5* gene showed hypomethylation in poor MPM survivors, with unadjusted *p* value < 0.05 (Table 1); instead, no CpGs in the *FKBP5* gene showed statistically significant hypermethylation in poor MPM survivors.

### 2.2. Survival Analysis

CpG sites and LMR were considered as predictors in the regression model. Categorical variables (quantile information) were used.

**Figure 2:**
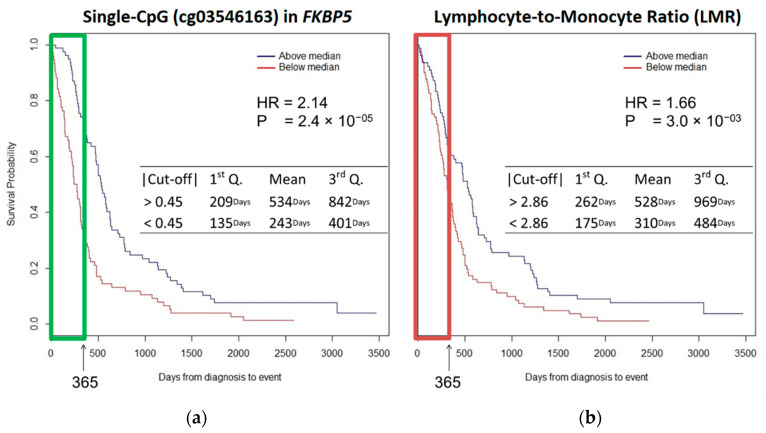
K-M survival curves show (**a**) cg03546163: patients with a DNAm < 0.45 had significantly poor survival compared with a DNAm ≥ 0.45 (mean, 243 versus 534 days; *p* value < 0.001); (**b**) LMR: patients with values < 2.86 had significantly poor survival compared with patients with values ≥ 2.86 (mean, 310 versus 528 days; *p* value < 0.001). cg03546163 is an independent marker of prognosis in patients with MPM and performs better than LMR (HR_cg03546163_ = 2.14 vs HR_LMR_ = 1.66).

Cox model was computed considering the same list of covariates included in the EWAS. Patients with DNAm < 0.45 at the cg03546163 had significantly poorer survival compared with subjects with DNAm ≥ 0.45 (mean, 243 versus 534 days; *p* value < 0.001). Survival at the 1st and the 3rd Quartiles was 135 versus 209 days and 401 versus 842 days, respectively, comparing patients with single CpG DNAm < 0.45 with those with single CpG DNAm ≥ 0.45. The multivariate analysis showed that cg03546163 DNAm at *FKBP5* was independently associated with OS. Kaplan–Meier curves revealed that a decrease of methylation at cg03546163 (<0.45) was significantly associated with worse OS (HR = 2.14 *p* value < 0.0001) (Figure 2a).

Patients with LMR < 2.86 had significantly poorer survival compared with patients with LMR ≥ 2.86 (mean, 310 versus 528 days; *p* value < 0.001). Survival at 1st Quartile was 175 versus 262 days whereas at 3rd Quartile was 484 versus 969 days comparing patients with LMR < 2.86 with those with LMR > 2.86. LMR was independently associated with OS: Kaplan–Meier curves showed that decreased LMR (<2.86) was significantly associated with decreased OS (HR = 1.66; *p* value < 0.01) (Figure 2b).

Histological subtype (epithelioid versus non-epithelioid), smoking status (current, never, and former), and asbestos exposure showed no statistically significant results on survival.

### 2.3. Validation and Replication

The statistically significant association between cg03546163 DNAm and OS was confirmed in an independent sample of patients (replication) and using a different targeted DNAm analysis technique (validation). A sample of 133 MPM cases (58 low survivors and 75 high survivors) was recruited and stratified in low and high OS considering the same cut-off (365 days).

The same model used for the discovery phase was performed. Patients with below median OS had significantly lower DNAm at the cg03546163 compared with those with above median OS (mean, 188 versus 786 days; *p* value < 0.001). The 1st Quartile was 113 versus 482 days and the 3rd Quartile was 262 versus 862 days comparing patients with DNAm difference (reference above median OS, MD: −0.04, 95%CI: −0.07|−0.01, *p* value: 0.04) at the cg03546163. The multivariate analysis confirmed that cg03546163 DNAm at *FKBP5* was independently associated with OS.

## 3. Discussion

A growing number of studies reported on the identification of epigenetic prognostic biomarkers in several cancers [17,22].

This study focused on the exploration of epigenetic factors related to MPM survival in MPM incident cases from Piedmont (Italy), a region with a well-documented history of asbestos exposure [23].

More than 450k methylation sites were evaluated in DNA from whole blood looking for new insights related to overall survival in MPM. The main result was the hypomethylation of a single CpG (cg03546163) in the 5′ UTR region of the FKBP5 gene in patients with poorer survival compared to patients with longer survival; it also showed to be an independent marker of prognosis in MPM patients. This result was replicated in a different series of patients belonging to the same cohort using the Sequenom Quantitative DNAm analysis.

In general, a combination of epigenetic and clinical factors is under investigation in clinical prognosis and survival, including tumor histology, gender, hemoglobin level, platelet and white blood cell count, and lactate dehydrogenase level [24].

Recently, due to the important role of inflammation in the development of MPM, several studies investigated the effect of inflammation-based biomarkers on the prognosis [11,22]. We selected the LMR for the comparison because its performance was previously reported to be higher than other inflammation-based markers in MPM [25].

To validate the prognostic value of the observed CpG methylation site, we compared our result with the LMR score.

Kaplan–Meier survival curves for MPM patients highlighted cg03546163 methylation at FKBP5 gene as a prognostic factor superior to the LMR score.

The FKBP Prolyl Isomerase 5 (FKBP5), also known as FK506 binding protein 51 (FKBP51), is a member of the immunophilin protein family, which contributes to the immunoregulation and to the basic cellular processes involving protein folding and trafficking. Together with other members of the FKBPs family, this protein participates in transcriptional complexes and acts as a co-transcription factor.

Although no studies have investigated the methylation of *FKBP5* as prognostic factor in MPM, a growing number of whole-blood studies investigated its DNA methylation levels in order to explain the impact of environmental stress in the etiology and treatment of several diseases [26]. Interestingly, in a recent study on the Behcet’s disease (BD) hypomethylation in the 5′UTR region (including cg03546163) of FKBP5 characterized cases was demonstrated and it was strongly associated with high gene expression, suggesting a possible role of DNA methylation in the pathogenesis [27].

Other five single CpGs at FKBP5 showed hypomethylation in poor survivors: this evidence supports the potential overall contribution of *FKBP5* methylation on the patient classification by OS.

In several human cancer tissues, a relevant role for FKBP5 in sustaining cancer cell growth and aggressiveness has been documented. In particular, for glioma [28], prostate cancer and melanoma [29] a strict correlation between protein abundance and aggressiveness has been demonstrated.

Probably, the relationship between FKBP5 and tumor progression and aggressiveness, is represented by its implication in NF-kB and AKT signaling pathways, with key roles in tumorigenesis and response to antineoplastic chemotherapy [30].

Moreover, a well characterized antiapoptotic effect is mediated by NF-κB transcription factors and FKBP5 has documented antiapoptotic effects: recent studies hypothesized that FKBP5 could promote inflammation, by activating the master immune regulator NF-kB, after an epigenetic upregulation due to aging and stress [31,32].

Previous studies conducted on various cancer types, showed that upregulation of FKBP5 gene expression is associated with drug resistance [33]. In a study on an ovarian cancer cell line, the upregulation of FKBP5 increased the resistance to chemotherapeutic agents, whereas the gene silencing sensitized ovarian cancer cells to taxol [34]. In the present study we could not evaluate FKBP5 gene expression due to the lack of available RNA, which was not collected in the study. However, this should be further addressed and verified in future studies.

One study demonstrated that overexpression of FKBP5 increased the chemosensitivity through the AKT pathway [31]. A similar study supported this observation making FKBP5 an effective biomarker for sensitivity to chemotherapy; patient responses to chemotherapy may be determined by the variation in FKBP5 levels [35].

### Limitation of the Study

Being able to identify the direction of causality will greatly aid in determining the usefulness of epigenetic variation.

Leukocyte DNA methylation could mainly represent a nonspecific marker related to a general inflammatory status due to the presence of a tumor rather than a specific MPM biomarker and further studies should be carried out to support our findings.

As additional limitation, we had therapy information only for a small subset of patients and we could not test treatment-specific OS differences in relation to FKBP5 methylation levels.

## 4. Material and Methods

### 4.1. Study Population

Study subjects belong to a wider ongoing collaborative study on MPM, which is actively enrolling MPM cases in the municipalities of Casale Monferrato (Piedmont region, Italy), an area with an exceptionally high incidence of mesothelioma caused by widespread asbestos exposure for locals, both occupational and environmental, due to the asbestos-cement Eternit plant that was operational until 1986 [36]. Additional MPM cases were recruited in the main hospitals of the municipalities of Turin, Novara, and Alessandria (Piedmont region, Italy). The study included incident MPM cases diagnosed between 2000 and 2010 after histological and/or cytological confirmation of MPM diagnosis [37,38].

No peritoneal cases were considered with the aim to better identify epigenetics characteristics of MPM.

In the present study, 159 MPM cases belonging to a larger case–control study with genetic [10,39] and blood DNAm data [9] were selected according to the following criteria: (i) availability of good quality DNA at the time of the analyses and (ii) asbestos exposure above the background level, as defined in [40]. An additional 133 independent samples from the same cohort were included for the validation/replication analyses.

Descriptive information of MPM patients are shown in Table 2. Median survival (365 days) was used as cut-off value to stratify patients in high and low survivors.

No differences in categorical (center, gender, smoke, histotype) and continuous (asbestos exposure, WBCs composition) variables among low and high survivors were found.

Our study complies with the Declaration of Helsinki principles and conforms to ethical requirements. All volunteers signed an informed consent form at enrollment. The study protocol was approved by the Ethics Committee of the Italian Institute for Genomic Medicine (prot.n.CE-2015-GM-2, 30/10/2015, HUGEF, Turin, Italy).

### 4.2. Exposure Assessment

For all subjects, occupational history and lifestyle habits information were collected through interviewer-administered questionnaires filled out at enrollment during a face-to-face interview. Job titles were coded according to the International Standard Classification of Occupations [40] and according to the Statistical Classification of Economic Activities in the European Community.

Frequency, duration, and intensity of exposure were estimated, then a cumulative exposure index was computed. The evaluation of asbestos exposure (occupational, environmental, and domestic) was conducted by an experienced occupational epidemiologist. For the selection criteria and descriptive evaluation, asbestos exposure doses (fibers/mL years) were rank transformed to remove skewness.

### 4.3. Blood DNAm Analysis

Genomic DNA was extracted from whole blood collected in EDTA by an on-column DNA purification method (QIAamp DNA Blood Mini Kit, QIAGEN GmbH, Germany), according to manufacturer’s instructions. DNA integrity was checked by an electrophoretic run in standard TBE 0.5× buffer on a 1% low melting agarose gel (Sigma-Aldrich GmbH, Schnelldorf, Germany); DNA purity and concentration were assessed by a NanoDrop 8000 Spectrophotometer (Thermo Fisher Scientific Inc., Waltham, MA, USA). Five hundred nanograms of genomic DNA for each sample were bisulfite treated (EZ-96 DNA Methylation-Gold Kit, Zymo Research Corporation, Irvine, CA, USA) to convert un-methylated cytosine to uracil. Cases were randomly and blindly distributed across conversion plates.

The Infinium HumanMethylation450 BeadChip (Illumina Inc., San Diego, CA, USA) was used to measure the methylation level of more than 485,000 individual CpG loci at a genome-wide resolution [41].

Twelve samples were analyzed on each BeadChip. As a “position effect” was reported for Illumina Methylation BeadChips, each sample position on the BeadChip was completely random as well. We further verified the randomization of the position on each BeadChip was effective by checking for a position effect, and we found no occurrence of it. BeadChips were processed according to manufacturer protocols. Data were inspected with the dedicated GenomeStudio software v2011.1 with Methylation module 1.9.0 (Illumina Inc., San Diego, CA), and quality checked as previously described [42].

### 4.4. Beta-Value Extraction

Raw DNAm data were analyzed with the R package (methylumi’). The average methylation value at each locus was computed as the ratio of the intensity of the methylated signal over the total signal (un-methylated + methylated) [43]. Beta-values represent the percentage of methylation at each individual CpG locus, ranging from 0 (no methylation) to 1 (full methylation).

We excluded from the analyses (i) single Beta-values with detection *p* value ≥ 0.01; (ii) CpG loci with missing Beta-values in more than 20% of the assayed samples; (iii) CpG loci detected by probes containing SNPs with MAF ≥ 0.05 in the CEPH (Utah residents with ancestry from northern and western Europe, CEU) population; (iv) samples with a global call rate ≤ 95%. Lastly, CpGs on chromosomes X and Y were excluded from the analysis.

### 4.5. Batch Effect, Population Stratification, and White Blood Cell Estimations

To account for methylation assay variability and batch effects, we corrected all differential methylation analyses for “control probes” principal components (PCs). Using PCs assessed by principal component analysis of the BeadChip’s built-in control probes as a correction factor for statistical analyses of microarray data is a method that allows to account for the technical variability of several steps in the DNAm analysis, from the bisulfite conversion to BeadChip processing [44].

Geographic origins of subjects may influence DNAm profiles. To consider this source of potential bias, we took advantage of the whole genome genotyping dataset from the same subjects from our previous study [10]. The first PCs calculated based on genome-wide genotyping were shown to correlate with different geographic origins of people [45,46].

WBC subtype percentages calculated based on genome-wide methylation data [47] for each subject were extracted. This method quantifies the normally mixed composition of leukocytes beyond what is possible by simple histological or flow cytometric assessments. In a diverse array of diseases and following numerous immune-toxic exposures, leukocyte composition will critically inform the underlying immune-biology to most chronic medical conditions. Then, it is necessary to extract and control for the percentage of involved WBCs with the aim to infer about a functional biological pathway.

LMR score was calculated from the DNAm-estimated WBCs by dividing the total lymphocyte count by the monocyte count.

### 4.6. Statistical Analyses

#### Epigenome-Wide Association Study

Association test was used to analyze the mean differences (MD) at single-CpG methylation between low and high survival. Multiple regression analysis adjusted for age, gender, histological subtype, asbestos exposure, smoke, estimated WBCs, population stratification (first 2 PCs) and technical variability (first 10 PCs) was implemented. For multiple comparisons tests, Bonferroni *p* value ≤ 0.05 was considered statistically significant.

Using random sampling methods, bootstrap was implemented to estimate the measures of accuracy defined in terms of bias, variance, confidence intervals, and prediction error. Bootstrap is also an appropriate way to control and check the stability of the results. The bias-corrected and accelerated (BCa) bootstrap interval was calculated with regard to single CpGs.

### 4.7. Survival Analysis

The survival time was determined as the time between the date of diagnosis and the date of death. If patients were still alive at the last follow-up (2016), survival was defined as the time from the date of diagnosis until June 2016. The time and the median event times with 95% confidence intervals were estimated according to the Kaplan–Meier method. The proportional hazards regression model was used for both the univariate and multivariate analyses (Cox model).

Comparison of OS curves was performed using two-tailed log-rank tests with a 0.05 level of significance. Only variables with *p* value < 0.1 in the univariate analysis were included in the final model for the multivariate analysis. In the Cox regression analysis, the backward conditional method (stepwise-AIC) was used. LMR and CpG sites were considered as predictors in regression model.

### 4.8. Statistical Power

To ensure a power of the study greater than 80% (two-tailed test at 0.05 alpha error), only CpGs with mean difference (MD) of Beta-value between low and high survival of ≥ |0.035| were selected. Covariates were included step-by-step in sensitivity analysis to validate the association output considering effect size, standard error, 95% confidence interval and *p* value variations.

CpGs with Bonferroni *p* value ≤ 0.05 underwent gene set enrichment analysis to identify pathways potentially affected by MPM related methylation changes.

All statistical analyses were conducted using the open source software R (4.0.2).

### 4.9. Validation and Replication

Sequenom MassARRAY for the DNAm signal validation and replication was used. In detail, the EpiTYPER assay (Sequenom) uses a MALDI-TOF mass spectrometry-based method to quantitatively assess the DNA methylation state of CpG sites of interest [48]. DNA (500 ng) was bisulfite-converted using the EZ-96 DNA Methylation Kit (Zymo Research) with the following modifications: incubation in CT buffer for 21 cycles of 15 min at 55 °C and 30 s at 95 °C, elution of bisulfite-treated DNA in 100 μL of water. The treatment converts unmethylated Cytosine into Uracil, leaving methylated Cytosine unchanged. In this way, variations in the sequence are produced depending on DNA methylation status of the original DNA molecule.

PCR amplification, treatment with SAP solution, and Transcription/RNase A cocktails were performed according to the protocol provided by Sequenom and the mass spectra were analyzed by EpiTYPER analyzer (Sequenom, San Diego, CA, USA). As the MassARRAY assay is unable to discriminate between CpGs located at close vicinity to each other in the sequence, the close neighboring CpGs were analyzed as “Units”, i.e., the measured methylation level is the average of the methylation levels of the CpGs cumulatively analyzed within the Unit. In the case of cg03546163 the measured methylation level is the average between two CpG sites located very close (Figure S1).

The amplicon for cg03546163 (chr6:35,654,364) encompasses 196bp (chr6:35,654,222-chr6:35,654,418 (GRCh37/hg19)) and PCR was performed on 10 ng of converted DNA using the following primers:

- cg03546163_10FW: aggaagagagTTTTTGTTTAGGATGAATTAGTTTGG;
- cg03546163_T7RV: cagtaatacgactcactatagggagaaggctAAAAACTACAATCTTATCCAATTCCTTT.

## 5. Conclusions

Our results suggest the potential use of DNAm analysis in blood to develop noninvasive tests for prognostic evaluation in MPM; our study is the first to demonstrate that a single CpG in *FKBP5* gene is an independent marker of prognosis in patients with MPM and is superior to the LMR inflammation-based prognostic score. The identification of simple and valuable prognostic markers for MPM will enable clinicians to select patients who are most likely to benefit from aggressive therapies and avoid subjecting nonresponder patients to ineffective treatment. Moreover, epigenetic modifications such as DNAm are potentially reversible and can open new perspectives for epigenetic therapies in MPM. Knowledge of epigenetic changes has provided new therapeutic opportunities against cancer. To allow better approach of cancer cell inhibitory strategies, the understanding of molecular mechanisms that underlie cellular DNA epigenetic alterations may be useful. In this context, we reported epigenetic deregulations in blood samples from MPM patients in relation to OS, paving the road to both patients’ stratification and the possible discovery of new combined therapeutic options in MPM. Studies of a large population are needed to investigate the relationship between prognostic markers and treatment regimens. The usage of methylation alterations in clinical specimens as biomarkers could be recognized. Noninvasively obtained, methylation-based biomarkers detected in blood cells from cancer patients offer significant practical advantages, being promising and dynamic prognostic markers.

## Figures and Tables

**Table 1:** Differential DNAm analyses of the *FKBP5* gene ordered by effect size (low survival group was used as reference). Information about single CpGs including location-related values and model outputs (effect size, standard error, *p* values).

TargetID	CHR	UCSC RefGene Group	Enhancer	Probe Start	Probe End	Closest TSS	Distance Closest TSS	Closest TSS Gene Name	Effect Size	SE	*p* value	Bonferroni	Significance
cg03546163	6	5′UTR;5′UTR;5’UTR;5′UTR	NA	35654313	35654363	35656691	2329	FKBP5	0.12	0.02	7.71E-09	0.003280418	*§
cg00052684	6	5′UTR	TRUE	35694195	35694245	35696396	2152	FKBP5	0.04	0.02	0.014589031	1	*
cg00130530	6	5′UTR;TSS1500;TSS1500;TSS1500	NA	35657152	35657202	35656718	−483	FKBP5	0.03	0.01	0.001490825	1	*
cg19226017	6	TSS1500;Body	NA	35697185	35697235	35696396	−788	FKBP5	0.03	0.01	0.021639194	1	*
cg08915438	6	TSS1500;Body	NA	35697709	35697759	35696396	−1362	FKBP5	0.02	0.01	0.050779639	1	*
cg14642437	6	5′UTR;5′UTR;5′UTR;5’UTR	NA	35652471	35652521	35656691	4171	FKBP5	0.02	0.01	0.030718193	1	*
cg25114611	6	TSS1500;Body	NA	35696820	35696870	35696396	−473	FKBP5	0.02	0.01	0.080435168	1	
cg16052510	6	Body;Body;Body;Body	TRUE	35603093	35603143	35656691	53549	FKBP5	0.01	0.01	0.201783727	1	
cg03591753	6	5′UTR	NA	35659141	35659191	35656718	−2422	FKBP5	0.01	0.01	0.071287867	1	
cg23416081	6	5′UTR	TRUE	35693573	35693623	35696396	2824	FKBP5	0.01	0.01	0.300181524	1	
cg19014730	6	5′UTR;5′UTR;5′UTR;5′UTR	TRUE	35635985	35636035	35656691	20707	FKBP5	0.01	0.01	0.510924063	1	
cg20813374	6	5′UTR;TSS1500;TSS1500;TSS1500	NA	35657130	35657180	35656718	−461	FKBP5	0.01	0.01	0.538622493	1	
cg07061368	6	5′UTR;5′UTR;5′UTR;5′UTR	TRUE	35631736	35631786	35656691	24956	FKBP5	0.00	0.01	0.440719926	1	
cg08636224	6	5′UTR;TSS1500;TSS1500;TSS1500	NA	35657871	35657921	35656718	−1202	FKBP5	0.00	0.00	0.18248273	1	
cg01294490	6	TSS200;TSS200;5′UTR;TSS1500	NA	35656906	35656956	35656718	−187	FKBP5	0.00	0.01	0.421300242	1	
cg07485685	6	5′UTR;Body	NA	35696060	35696110	35696396	336	FKBP5	0.00	0.00	0.847941933	1	
cg14284211	6	Body;Body;Body;Body	TRUE	35570224	35570274	35656691	86468	FKBP5	0.00	0.01	0.974344781	1	
cg17030679	6	5′UTR;Body;1stExon	NA	35696300	35696350	35696396	97	FKBP5	0.00	0.00	0.955719442	1	
cg00862770	6	5′UTR;5′UTR;5′UTR;5′UTR	NA	35655764	35655814	35656691	928	FKBP5	0.00	0.00	0.939904147	1	
cg00140191	6	5′UTR;5′UTR;5′UTR;5′UTR	NA	35656193	35656243	35656691	450	FKBP5	0.00	0.00	0.882388191	1	
cg00610228	6	5′UTR;Body	NA	35695934	35695984	35696396	463	FKBP5	0.00	0.00	0.87376216	1	
cg07633853	6	Body;Body;Body;Body	TRUE	35569421	35569471	35656691	87221	FKBP5	0.00	0.01	0.965427693	1	
cg10300814	6	Body;Body;Body;Body	TRUE	35565066	35565116	35480646	−84469	TULP1	0.00	0.00	0.620677997	1	
cg16012111	6	TSS200;TSS200;TSS200;5′UTR	NA	35656758	35656808	35656718	−39	FKBP5	0.00	0.00	0.519047184	1	
cg06937024	6	5′UTR;Body	NA	35695440	35695490	35696396	908	FKBP5	0.00	0.00	0.135004544	1	
cg08586216	6	5′UTR;5′UTR;5′UTR;5′UTR	TRUE	35612301	35612351	35656691	44341	FKBP5	0.00	0.00	0.105631333	1	
cg17085721	6	5′UTR;5′UTR;5′UTR;5′UTR	TRUE	35645291	35645341	35656691	11351	FKBP5	0.00	0.00	0.211582562	1	
cg02665568	6	Body;Body;Body	NA	35544468	35544518	35480646	−63821	TULP1	−0.01	0.01	0.294757699	1	
cg15929276	6	5′UTR	TRUE	35687456	35687506	35696396	8940	FKBP5	−0.01	0.01	0.455969031	1	
cg06087101	6	Body;3′UTR;Body;Body	NA	35551882	35551932	35480646	−71285	TULP1	−0.02	0.02	0.203783874	1	

Low survival group was set as reference. Adjustment covariates: age, gender, asbestos exposure, histological subtype, smoke, population stratification, WBCs estimation, and technical variability. *: statistically significant at *p* value< 0.05; §: statistically significant at Bonferroni and FDR post hoc adjustments.

**Table 2:** Descriptive information of MPM patients. Median survival (365 days) was used as cut-off value to stratify patients in high and low survivors.

**Categorical Variable**	**Level**	**Low OS (*n* = 79)**		**High OS (*n* = 80)**	
		N	%	N	%
Centre	Casale	50	63.3	46	57.5
	Torino	29	36.7	34	42.5
Gender	Males	59	74.7	50	62.5
	Females	20	25.3	30	37.5
Smoke	Current	20	26.3	8	10.3
	Former	24	31.6	29	37.2
	Never	32	42.1	41	52.6
Histotype	Epithelioid	44	55.7	61	76.3
	Sarcomatoid	14	17.7	2	2.5
	Biphasic	17	21.5	11	13.8
	Undefined	2	2.5	1	1.3
	Not known	2	2.5	5	6.3
**Continuous Variable**	**Level**	**Low OS**		**High OS**	
		Mean	SD	Mean	SD
Overall Survival (days)		198.7	101.6	957.8	698.7
Age (years)		67.7	12.4	67.5	9.6
Asbestos Exp. (norm)		1.4	1.5	1.5	1.9
CD8T (%)		2.9	4.5	3	3.4
CD4T (%)		6.8	5.3	8.8	5.4
Natural Killer (%)		4.9	4.9	6.3	4.1
B cell (%)		6.1	2.8	6.4	2.7
Monocytes (%)		8.1	4.1	7.6	4.4
Granulocytes (%)		75	13	72	10

Asbestos exposure (occupational, environmental, and domestic) was normalized considering frequency, duration, and intensity.

## Supplementary Materials

The following are available online at https://www.mdpi.com/2072-6694/12/11/3470/s1, Figure S1. Locations of cg03546163 (CpG2, in bold) and a second CpG site very close (CpG1, in red), investigated by Sequenom MassARRAY.
