# Supplementary material for: DNA Methylation of FKBP5 as Predictor of Overall Survival in Malignant Pleural Mesothelioma
Source: Cancers (Basel). 2020 Nov 21;12(11):3470. doi: 10.3390/cancers12113470 (PMC7700347; doi:10.3390/cancers12113470)
Supplement: Supplementary file 1 [file cancers-12-03470-s001.pdf]

cancers

Supplementary Materials

DNA methylation of *FKBP5* as predictor of overall survival in Malignant Pleural Mesothelioma

Giovanni Cugliari, Chiara Catalano, Simonetta Guarrera, Alessandra Allione, Elisabetta Casalone, Alessia Russo, Federica Grosso, Daniela Ferrante, Clara Viberti, Anna Aspesi, Marika Sculco, Chiara Pirazzini, Roberta Libener, Dario Mirabelli, Corrado Magnan, Irma Dianzani, Giuseppe Matullo

cg03546163

TTTTTGTTTAGGATGAATTAGTTTGGAGAGGAAAGAATATTTTGTTTGTATATTTGT
AGAAGAAGTTATTGTTGTTAAAAGTTAAGTTGGTTTTTTGATGTTTAGTGTGGATAG
GTTGAATAATAATTTATTTTTA**CG**(CpG1)TT**CG**(CpG2)GAGGGTTTATTTTATGTA
AATAGTTGAAAGGAATTGGATAAGATTGTAGTTTTT

Figure S1. Locations of cg03546163 (CpG2, in bold) and a second CpG site very close (CpG1, in red), investigated by Sequenom MassARRAY. Flanking primers are underlined. The CpG sites could not be tested individually due to MassARRAY technology constraints, but had to be tested jointly as a single unit: the methylation level is the cumulative value of all the sites within the CpG unit.

© 2020. by the authors. Licensee MDPI, Basel, Switzerland. This article is an open access article distributed under the terms and conditions of the Creative Commons Attribution (CC BY) license (http://creativecommons.org/licenses/by/4.0/).
